# Morphological specializations of mosquito CO_2_-sensing olfactory receptor neurons

**DOI:** 10.1073/pnas.2514666122

**Published:** 2025-10-23

**Authors:** Shadi Charara, Jonathan Choy, Kalyani Cauwenberghs, Pawel Vijayakumar, Renny Ng, Keun-Young Kim, Shih-Che Weng, Omar S. Akbari, Mark H. Ellisman, Scott A. Rifkin, Chih-Ying Su

**Affiliations:** ^a^Department of Neurobiology, University of California San Diego, La Jolla, CA 92093; ^b^National Center for Microscopy and Imaging Research, Center for Research in Biological Systems, University of California San Diego, La Jolla, CA 92093; ^c^Department of Cell and Developmental Biology, University of California San Diego, La Jolla, CA 92093; ^d^Department of Ecology, Behavior, and Evolution, University of California San Diego, La Jolla, CA 92093

**Keywords:** CO_2_-sensing olfactory receptor neuron, mosquito, serial block-face scanning electron microscopy, morphometrics, nonsynaptic axonal varicosity

## Abstract

Hematophagous mosquitoes use CO_2_ as a key arousal signal that gates behavioral responses to host-derived cues. In *Aedes aegypti*, CO_2_ is detected by olfactory receptor neurons (ORNs) housed in the sensory hairs (sensilla) on the maxillary palp. While the molecular mechanism and behavioral significance of CO_2_ sensing have been well studied in mosquitoes, the nanoscale three-dimensional structures of their CO_2_-sensing ORNs and associated cells have remained unclear. Using serial block-face scanning electron microscopy, we characterize the CO_2_-sensing cpA neuron and its odor-sensitive neighbors, cpB and cpC, within the capitate sensilla of *A. aegypti.* Notably, cpA neurons are significantly larger, with an outer dendritic surface area 8 to 12 times greater than that of cpB and cpC neurons. This expanded CO_2_-sensing surface arises from its unique architecture, consisting of numerous flattened dendritic sheets folded into intricate lamellae. In contrast, cpB and cpC dendrites exhibit sparse, narrow cylindrical branches. Moreover, the cpA axon displays a prominent pearls-on-a-string morphology, with numerous mitochondria-rich, nonsynaptic varicosities connected by thin cables. Remarkably, a glial cell and an auxiliary cell together ensheathe the cpA soma but not cpB or cpC, suggesting a specialized role in supporting cpA function. Compared to *Drosophila* CO_2_-sensitive ORNs, a larger portion of the cpA outer dendrite is embedded within the sensillum cuticle, potentially improving access to environmental CO_2_. These findings reveal key morphological specializations of cpA neurons, thereby advancing our understanding of mosquito sensory biology and laying the groundwork for future studies on the molecular basis and functional ramifications of these anatomical adaptations.

## Significance

Carbon dioxide (CO_2_) emitted by human hosts is a critical cue that mosquitoes use for host detection, yet the nanoscale three-dimensional (3D) structure of their CO_2_-sensing neurons and associated cells remains unclear. Elucidating the anatomy of these cells will yield structural insight into the sensory biology which drives mosquito−host interactions. Using volume electron microscopy, we reveal that *Aedes aegypti* CO_2_-sensing neurons exhibit striking structural specializations—including enlarged CO_2_-sensing surface areas, unique axonal architecture enriched with mitochondria, and unusual somatic ensheathing by support and glial cells—that likely enhance CO_2_ detection and support signal transmission. Our detailed anatomical characterization provides a structural basis for the mosquito’s exceptional host-seeking capabilities.

Insects rely on olfactory cues to forage, seek mates, and avoid predators (1). Among behaviorally significant odorants, carbon dioxide (CO_2_)—a highly volatile byproduct of respiration and fermentation—holds particular ethological importance (2, 3). For instance, hematophagous mosquitoes use CO_2_ as a key arousal cue, gating behavioral responses to a wide range of other host-derived signals (4). Similarly, other blood-sucking insects, such as tsetse flies and sandflies, are strongly attracted to CO_2_ (2). At the molecular level, the primary mosquito CO_2_ receptor is a heteromeric complex formed by members of a highly conserved gustatory receptor subfamily (5). In mosquitoes, CO_2_ detection occurs in olfactory receptor neurons (ORNs) located on the maxillary palp, and is mediated by three gustatory receptors: Gr1, Gr2, and Gr3 in the yellow fever mosquito *Aedes aegypti* or Gr22, Gr23, and Gr24 in the malaria vector *Anopheles gambiae* (4–7). Targeted deletion of these gustatory receptors impairs mosquitoes’ CO_2_-mediated host-seeking behavior (4).

Most insect olfactory sensory hairs, or sensilla, house multiple ORNs, each assigned a cellular identity based on the host sensillum and its relative extracellular spike amplitude (8). Typically, two to four ORNs are housed together in stereotyped combinations, wherein neurons expressing certain receptors are consistently paired together in a genetically determined manner (9–13). For example, in *A. aegypti*, the CO_2_-sensing *Gr1*, *Gr2*, and *Gr3* receptors are coexpressed in the large-spike “A” neuron housed within the capitate peg sensillum (cpA). This neuron is paired with two small-spike odor-sensing neighbors expressing the *AaegOr8* and *AaegOr49* receptors, respectively (4, 14–17).

While the molecular mechanism and behavioral significance of mosquito CO_2_-sensing neurons have been extensively studied, their three-dimensional (3D) morphology and nanoscale morphometrics remain unknown. This information is important because odor sensitivity is thought to scale with the size of sensory surface area (18, 19). For example, in the *Manduca* moth, the long ORN dendrites likely contribute to the insect’s high pheromone acuity (20–23). In addition, the size differences between insect ORNs housed within the same sensillum influence the relative strength of their direct electrical neuronal interactions, known as ephaptic coupling (24–26). Computational models further suggest that the degree of size disparity between cohoused ORNs determines the sensillum’s optimal odor mixture ratio, which is expected to elicit the most robust behavioral response (24).

Evidence indicates that insect CO_2_-sensing neurons exhibit unique morphological adaptations. We recently showed that the CO_2_-sensing ab1C ORNs of *Drosophila melanogaster* have flattened, sheet-like dendrites (27)—which contrast sharply with the cylindrical dendritic branches typical of odor-sensing neurons (28). Moreover, transmission electron microscopy (TEM) studies of capitate peg sensilla in *A. aegypti* and *A. gambiae* suggest that the sensory dendrites of mosquito CO_2_-sensing neurons contain folded lamellae (7, 29, 30). However, without genetic labeling or other specific identification methods, it is unclear whether these lamellated dendrites are indeed associated with CO_2_-sensing neurons. Furthermore, the three-dimensional (3D) structure of these dendritic lamellae remains undefined, as the transmission electron microscopy (TEM) studies provide primarily two-dimensional (2D) snapshots of neuronal morphology.

To address these questions, here we generated a serial block-face scanning electron microscopy (SBEM) volume of the maxillary palp of *A. aegypti*. The tissues were prepared using the CryoChem method, which we previously developed to ensure high-quality ultrastructural preservation of cryofixed samples while allowing for adequate *en bloc* heavy metal staining required for volume electron microscopy (EM) (31). High-pressure freezing followed by freeze-substitution is essential for effectively preserving tissues with cuticles (e.g., insect sensory appendages), which are impermeable to chemical fixatives. Importantly, cryofixation provides superior morphological integrity, whereas chemical fixation can cause membrane distortion that compromises accurate morphometrics quantification (31–33).

Importantly, our previous studies using genetic labeling of multiple identified *Drosophila* ORNs in SBEM volumes demonstrated that within a sensillum, the rank order of ORN soma sizes corresponds to their relative extracellular spike amplitudes. This relationship arises from the size-dependent electrotonic properties of neurons, whereby larger neurons exhibit lower input resistance. As a result, compartmentalized ORNs can be classified as “A,” “B,” or “C” types in descending order of spike amplitude (26, 28, 31). Although this principle was established in *Drosophila*, we reason that it also applies to mosquitoes ORNs due to the conserved biophysical relationship between neuronal size and electrotonic properties across species. Moreover, because capitate sensilla are the only olfactory sensilla located on the mosquito maxillary palp (29, 30), the cp sensilla can be readily identified, and the identities of cpA, B, or C neurons can be assigned based on their relative neuronal sizes without requiring genetic labeling.

Using volume EM technology to reconstruct the 3D structures of mosquito CO_2_-sensing neurons, our study revealed key morphological specializations that likely enhance CO_2_ detection and support cpA-specific signal conduction and physiology. Comparative analysis with fruit fly CO_2_-sensing neurons further identified species-specific adaptations, including greater sensillum cuticle encapsulation of the mosquito cpA dendrite and a dedicated glial cell and a unique tormogen cell that ensheathes the cpA soma but not cpB or cpC. These findings offer structural insight into the sensory biology driving mosquito−host interactions.

## Results

**Fig. 1.**
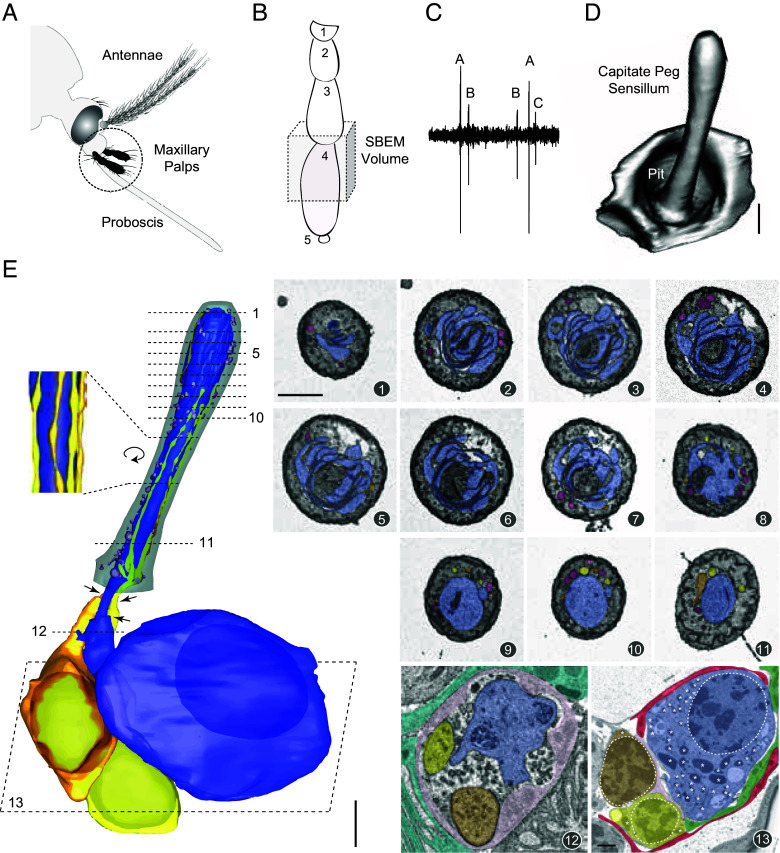
Identification of mosquito capitate peg ORNs in a SBEM volume. (*A*) Chemosensory appendages of a female *A. aegypti*, with the maxillary palps highlighted in black (Adapted from ref. 37). (*B*) Schematic of the five maxillary palp segments, with the distal end at the *Bottom*. The imaged region is boxed in gray, containing the fourth segment where cp sensilla are located. (*C*) Representative single-sensillum recording trace of the spontaneous spike activities, demonstrating the distinct extracellular spike amplitudes of the three ORNs (*A*–*C*) housed in the cp sensillum. (*D*) 3D model of a cp sensillum in a pit-like indentation. (Scale bar: 2 µm.) (*E*) 3D models and representative SBEM images of a cp sensillum and its ORNs. The sensillum cuticle (gray) is shown from the base to the tip. Dashed lines mark SBEM image positions: (1–8) distal outer dendrites, highlighting cpA’s intricate dendritic lamellae; (9–11) proximal outer dendrites; (12) inner dendrites; and (13) ORN somata with nuclei (outlined) and mitochondria (*). Cells are pseudocolored: cpA (blue), cpB (orange), cpC (yellow), thecogen (pink), trichogen (turquoise), tormogen (green), and glial cell (crimson). Extracellular vesicles are pseudocolored in aubergine. Arrows mark ciliary constrictions, which separate the inner and outer dendritic segments. (*Inset*) Magnified and rotated view, highlighting the morphology of cpB and cpC dendritic branches. Scale bars: 2 μm for 3D models and 1 μm for SBEM images. The first SBEM scale bar applies to all images unless indicated otherwise. See also Movie S1, corresponding to the first sensillum model.

To determine the 3D structure of mosquito CO_2_-sensing neurons, we generated a serial SBEM volume of an *A. aegypti* maxillary palp (Fig. 1*A*). The SBEM images data have been deposited in Cell Image Library (CIL:57520) (34). Mosquito CO_2_-sensing neurons reside in capitate peg (cp) sensilla on the fourth maxillary palp segment (Fig. 1*B*) (29, 30, 35). Within the SBEM volume, cp sensilla can be unambiguously identified because they are the only olfactory sensilla on the maxillary palp that contain three neurons (Fig. 1*C*). These sensilla are distinguished by their club-shaped cuticle located within a pit-like indentation (Fig. 1*D*) (29, 30, 35, 36). Based on the 3D models of cp sensilla, we determined their average dimensions as 12.98 ± 0.69 µm in length, 84.57 ± 3.06 µm^2^ in surface area, and 35.16 ± 1.48 µm^3^ in volume (mean ± SEM, *n* = 5; see *SI Appendix*, Table S1 for raw data), similar to the size of large basiconic sensilla in *Drosophila* (28).

From the palp SBEM volume, we generated 3D models of cp ORNs (Fig. 1 *E*, *Left*). Similar to *Drosophila* antennal ORNs (28), the cell bodies and inner dendrites of *Aedes* ORNs were located beneath the base of the sensillum cuticle. Outer dendrites extended into the sensillum lumen from ciliary constrictions, which separate the inner and outer dendritic segments (32). Numerous extracellular vesicles were also observed in the lumen (Fig. 1*E*), corresponding to the luminal vacuoles reported in *Drosophila* olfactory sensilla (28).

### Morphological Characterization of Mosquito cp ORNs.

Among the three neurons in the cp sensillum, the CO_2_-sensing cpA ORN was identified as the largest neuron, while the odor-sensing cpB and cpC neurons were the intermediate and smallest, respectively (Fig. 1*E*). As postulated in earlier TEM studies (7, 29, 30), the lamellated dendrites were indeed associated with the cpA neuron. Intricate dendritic lamellae of varying thickness were observed in the distal region of the cpA outer dendrite (Fig. 1*E*, Images 1 to 8), spanning approximately one-third of the sensillum length and corresponding to the bulging region of the club-shaped cuticle. In contrast, the proximal cpA outer dendrite was thick and trunk-like, with a diameter nearing 1 µm (Fig. 1*E*, Images 9 to 11). These features contrast sharply with those of the neighboring cpB and cpC neurons, whose outer dendrites consist of sparse, narrow cylindrical branches decorated with small, periodic beady structures (Fig. 1 *E*, *Inset* and Movie S1), resembling odor-sensing ORNs in fruit flies and silkmoth (27, 28, 38, 39).

Surprisingly, the inner dendritic segment of the cpA neuron was neither enlarged nor densely packed with mitochondria (Fig. 1*E*, Image 12), unlike the large-spike “A” neurons housed in the large basiconic sensilla of *Drosophila* (28). Instead, mitochondria were concentrated around the nucleus within the cpA soma (Fig. 1*E*, Image 13).

In *Drosophila* ORNs, the enlarged inner dendritic “mitochondrial pack” is thought to support the metabolic demands of complex dendritic structures or extensive sensory surface areas. Additionally, the mitochondria-rich inner dendrite, which separates the outer dendritic compartment from the soma, likely limits the spread of dendritic Ca^2+^ influx into the soma, thereby acting as a spatial Ca^2+^ buffer (40). However, our findings in cpA neurons suggest that insect ORNs with complex outer dendrites do not necessarily require numerous inner dendritic mitochondria, raising intriguing questions about the relationship between inner dendritic mitochondria, outer dendritic size, and ORN-specific physiology.

### 3D Reconstruction Reveals Intricate cpA Dendritic Lamellae.

**Fig. 2.**
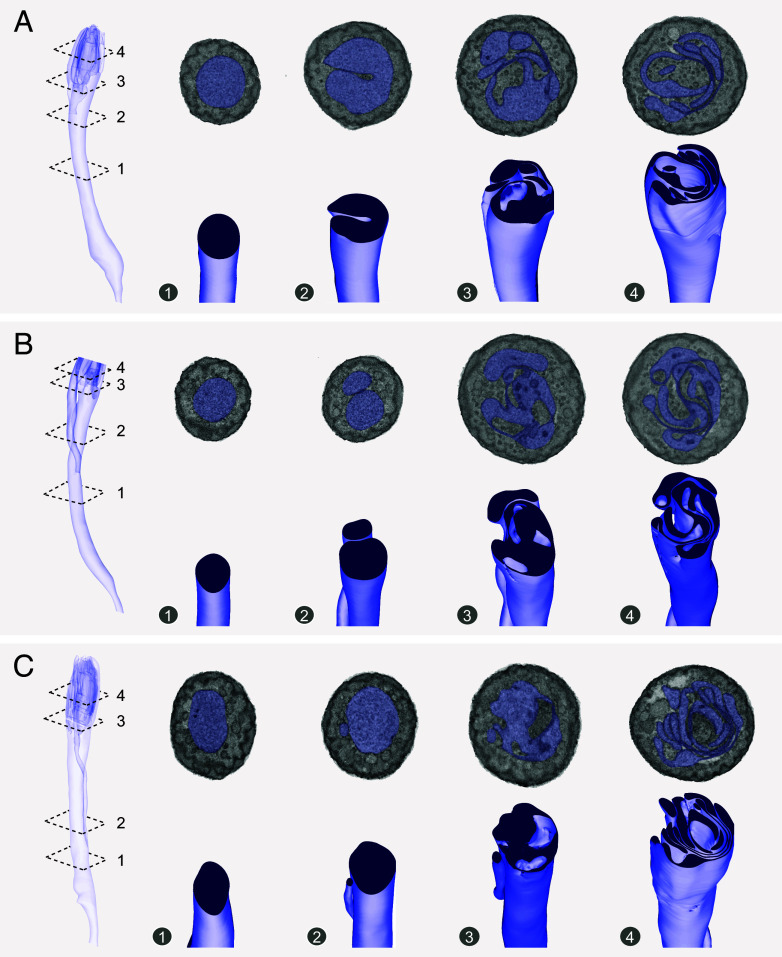
3D models reveal intricate cpA dendritic lamellae. (*A*) 3D model of a cpA outer dendrite. The ciliary constriction is shown at the narrowed end at the *Bottom*. Dashed planes indicate the positions of the corresponding cross-sectional SBEM images (*Top*) and clipped views (*Bottom*). The dendrite’s cytoplasm is colored in dark blue in the clipped 3D models. (*B* and *C*) Outer dendritic 3D models of two other cpA ORNs, highlighting their dendritic lamellae. The dendritic tip of the neuron in (*B*) was truncated during image acquisition. The neuron in (*C*) corresponds to the one shown in Fig. 1*E*.

3D models of three different cpA neurons were reconstructed from the palp SBEM volume, revealing unexpected complexity in the cpA outer dendrite (Fig. 2). At the distal end, we observed convoluted outer dendritic lamellae which extended from a thick, trunk-like proximal segment (Fig. 2*A*, Image 1) before folding inward on one side to present a kidney-shaped cross-sectional profile (Fig. 2*A*, Image 2). Further along, additional folds appeared, while existing ones deepened, forming multilayered lamellae with varying thickness (Fig. 2*A*, Images 3 to 4).

In two other cpA neurons, intricate lamellae formed from extensive membrane folding and minor branching (Fig. 2 *B* and *C*). Similar to the first neuron, their proximal segments exhibited a trunk-like morphology (Fig. 2 *B* and *C*, Image 1). However, around the outer dendritic mid-point, a side branch emerged (Fig. 2 *B* and *C*, Image 2), which became increasingly flattened toward the distal end to form a standalone lamella (Fig. 2 *B* and *C*, Images 3 to 4). Meanwhile, extensive membrane folding along the main dendritic trunk generated additional lamellae (Fig. 2 *B* and *C*, Images 3 to 4). Collectively, these 3D models also highlight structural heterogeneity among cpA outer dendrites, similar to the diverse dendritic morphologies observed among *Drosophila* CO_2_-sensing ab1C ORNs (27).

Unlike the structured, evenly spaced lamellated dendrites of insect thermosensitive neurons, which are interconnected by bossy orthogonal surface substructures [Bibr r41](41–44), cpA lamellae exhibited a more complex and irregular folding pattern. This distinct structural organization likely reflects varying molecular mechanisms during development and warrants further investigation in future research.

### Prominent Pearls-on-a-String Morphology of cpA Axon.

**Fig. 3.**
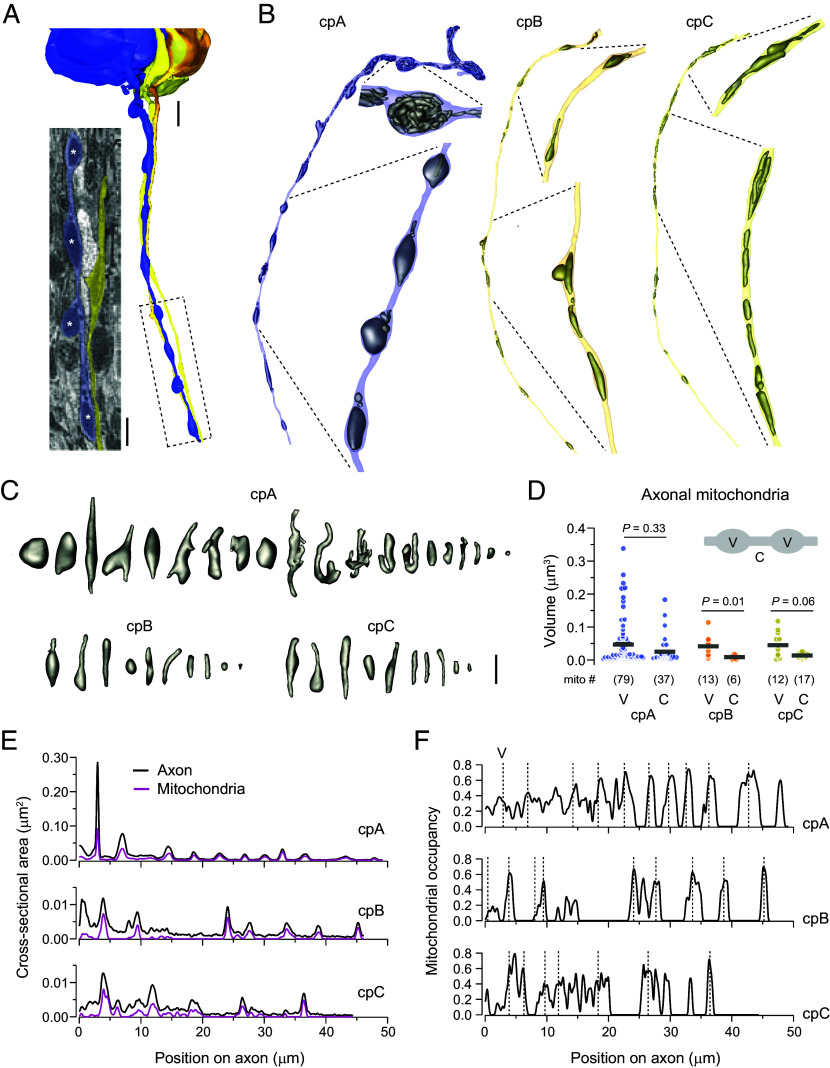
cpA axons exhibit a prominent pearls-on-a-string morphology. (*A*) 3D models of cp ORN axons, with neurons pseudocolored as follows: cpA (blue), cpB (orange), cpC (yellow). SBEM image: A longitudinal section of cpA and cpC axons (boxed region), rendered using the IMOD software. White asterisks indicate large mitochondria within cpA varicosities. Scale bars: 2 μm for 3D models and 1 μm for SBEM image. (*B*) 3D models of individual cp axons and their mitochondria, corresponding to those shown in panel (*A*). Axon hillocks are positioned at the *Top*. Enlarged views highlight axonal mitochondria. (*C*) Representative 3D models of mitochondria sampled from the axons shown in (*B*), arranged from left to right in descending order of volume. (Scale bar: 1 μm.) (*D*) Quantification of mitochondrial volumes from axons shown in (*B*). V: varicosity; C: connector. Gray bars indicate the mean value. The number of mitochondria is shown in parentheses. Statistical significance was assessed with the Mann–Whitney rank sum test. (*E*) Axon and total mitochondrial cross-sectional areas are plotted along the axon, relative to the axon hillock (position 0). (*F*) Mitochondrial occupancy, calculated as the ratio of mitochondrial to axon cross-sectional area at each axon position. Dashed lines indicate varicosity center positions. See also *SI Appendix*, Fig. S1 and Movie S2.

In addition to analyzing the somas and dendrites of cp ORNs, we examined their axonal morphology by segmenting ~45 µm of axon from the hillock onward. Surprisingly, cpA axons displayed a prominent pearls-on-a-string structure (Fig. 3*A*), with mitochondria-packed varicosities containing no synaptic vesicles. Notably, several cpA varicosities were filled by a single large mitochondrion (Fig. 3 *B*, *Left*). In contrast, cpB and cpC axons had smaller mitochondria-containing nonsynaptic varicosities and lacked the pronounced pearls-on-a-string morphology (Fig. 3 *B*, *Middle* and *Right*).

3D reconstruction of mitochondrial structure revealed diverse shapes—round, elongated, branched, and compact (Fig. 3*C*) (45)—with volumes ranging from 0.001 to 0.338 µm^3^. Within the segmented cpA axon, we identified 116 mitochondria: 79 in varicosities and 37 in regions between varicosities (connectors). The majority of cpA mitochondria were small (<0.05 µm^3^), with no significant volume difference between the varicosity and connector regions, although large mitochondria were more frequently found within varicosities. In comparison, the cpB and cpC axons contained fewer mitochondria—19 and 29, respectively—with a similar trend toward larger mitochondria in varicosities (Fig. 3*D*).

To determine the dimensions of varicosities and connectors, we measured axonal cross-sectional areas along each axon for the cp trio (Fig. 3*E*, black traces). On average, the cpA varicosities were larger than those in cpB and cpC. Given that the dimensions of cpB and cpC varicosities did not differ significantly, their measurements were combined for subsequent analyses.

We expanded our survey to include additional ORN axons in the same nerve fascicle (*SI Appendix*, Fig. S1*A*) and confirmed that cpA varicosities were both wider and longer than those in cpB/C (*SI Appendix*, Fig. S1 *B*–*D*). On the other hand, connector lengths varied considerably among the cp axons, although cpA connectors were significantly wider than those in cpB/C (*SI Appendix*, Fig. S1*E*). Varicosity density—measured as the number per unit axon length—was similar across cp neurons, averaging approximately 0.2 per µm (*SI Appendix*, Fig. S1*F* and Table S1).

To examine the relationship between mitochondria occupancy and axonal morphology, we also analyzed total mitochondrial cross-sectional area (Fig. 3*E*, magenta traces). Notably, its fluctuating profile mirrored that of varicosities and connectors, suggesting that the axonal morphology of cp ORNs is largely determined by mitochondrial content, unlike the varicosity in rat hippocampal axons which are determined by membrane mechanics (46). In support of this notion, mitochondrial occupancy tended to be higher at varicosities, with peak values reaching ~0.7 across all three cp ORNs (Fig. 3*F*).

### Auxiliary and Glial Cells Associated with cp ORNs.

Olfactory sensilla contain three auxiliary cell types—thecogen, trichogen, and tormogen—which are distinguished by their morphology and relative positions along the core-to-surface axis of the olfactory organ (28, 47). In *Drosophila* sensilla, the thecogen cell forms a tight sleeve around the outer dendrite beneath the cuticle base, entire inner dendrite, and a small part of the ORN soma. The trichogen cell, situated distal and lateral to the thecogen cell, surrounds the thecogen’s apical region and borders the sensillum-lymph cavity with extensive microlamellae. The tormogen cell, the outermost of the three, partially surrounds the trichogen cell and borders the cuticle surface (28). The somas of individual fly ORNs are tightly surrounded by thin glial sheaths (47) (*SI Appendix*, Fig. S2*A*), likely providing electrical insulation.

**Fig. 4.**
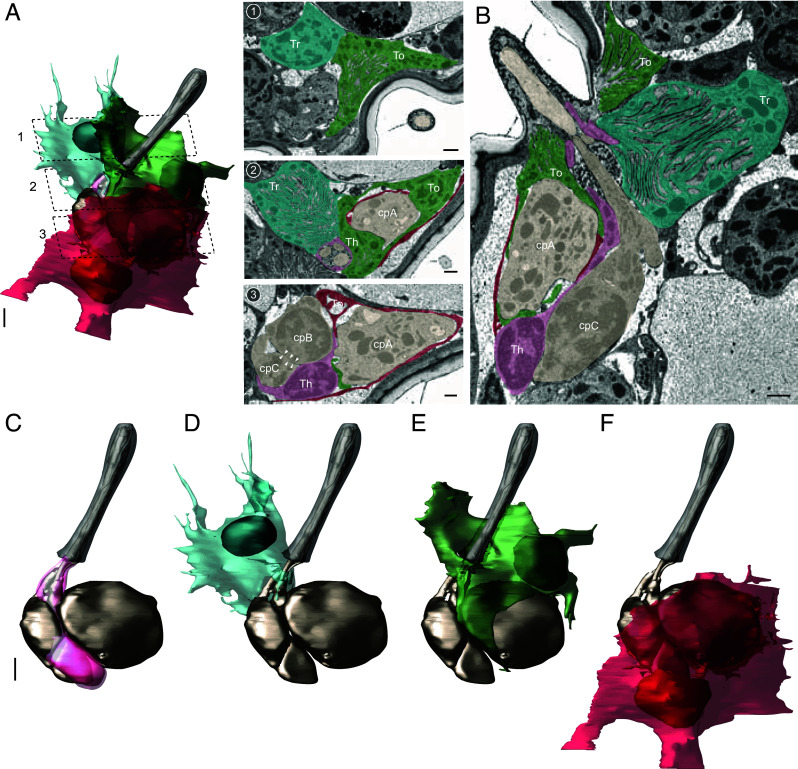
Auxiliary and glial cells associated with cp ORNs. (*A*) 3D model and SBEM images of a cp sensillum, showing its three auxiliary cells and one glial cell in relation to ORNs. Cells are pseudocolored to indicate identities: ORNs (bronze), thecogen cell (pink, Th), trichogen cell (turquoise, Tr), tormogen cell (green, To), and glial cell (crimson). Dashed lines mark positions of the corresponding SBEM images. White arrowheads indicate possible direct contacts between the somas of cpB and cpC. (*B*) A longitudinal sensillum section rendered by IMOD. (*C*–*F*) 3D models of individual auxiliary and glial cells: thecogen (*C*), trichogen (*D*), tormogen (*E*), and the cpA soma-ensheathing glial cell (*F*). For simplicity, microlamellae of trichogen and tormogen cells were not shown in 3D models. Scale bars: 2 μm for 3D models and 1 μm for SBEM images. See also *SI Appendix*, Figs. S2 and S3, and Movies S3 and S4.

In the mosquito cp sensillum, three auxiliary cells were organized similarly to those in flies (Fig. 4*A* and Movie S3). The thecogen cell closely enveloped the inner dendrite and the outer dendritic region beneath the cuticle base, and covered small parts of cpB and cpC somas (Fig. 4 *A*–*C*). Positioned distally and laterally to the thecogen cell, the trichogen cell extended numerous microlamellae into the basal lateral region of the sensillum-lymph cavity (Fig. 4 *B* and *D*). Further distal to the trichogen cell, the tormogen cell extended numerous microlamellae surrounding the sensillum-lymph cavity bordering the cuticle surface of maxillary palp (Fig. 4*B*).

Surprisingly, the cp tormogen cell exhibited an unusual morphology. Unlike *Drosophila* tormogen cells, which do not ensheathe ORNs (27, 28, 47), the mosquito tormogen cell selectively and tightly wrapped around the upper portion of the cpA soma near the dendrite (Fig. 4*E*, *SI Appendix*, Fig. S3, and Movie S4). In contrast, the lower half of the cpA soma was ensheathed by a dedicated glial cell that did not envelope cpB or cpC somas (Fig. 4*F*, *SI Appendix*, Fig. S3, and Movie S3). The processes of the tormogen and glial cells overlapped at their junctions: In some regions, the glial process laid on top of the tormogen process (*SI Appendix*, Fig. S3*A*), while in others, the tormogen process covered the glial process (*SI Appendix*, Fig. S3*B*). In a small area where the cpA soma was not enveloped by either cell, it was instead covered by the thecogen cell (*SI Appendix*, Fig. S3*C*). That is, the entire cpA soma was enveloped, primarily by the tormogen and glial cells, with a small region covered by the thecogen cell. This specialized cellular architecture may provide targeted insulation and functional support specifically to the cpA neuron.

In comparison, cpB and cpC somas appeared to have direct contacts (Fig. 4*A*, Image 3, white arrowheads), lacking intervening glial sheaths (Fig. 4*A* and *SI Appendix*, Fig. S2*B*). These soma-to-soma contacts may enable electrical (ephaptic) interactions between cpB and cpC beyond their outer dendritic regions, similar to what was postulated for clustered core neurons in the mouse suprachiasmatic nucleus (48). Future research should explore the functional significance of this selective tormogen and glial ensheathing of cpA soma, particularly in contrast to its exclusion from the neighboring cpB and cpC somas.

### Glial Cells Associated with cp Axons.

**Fig. 5.**
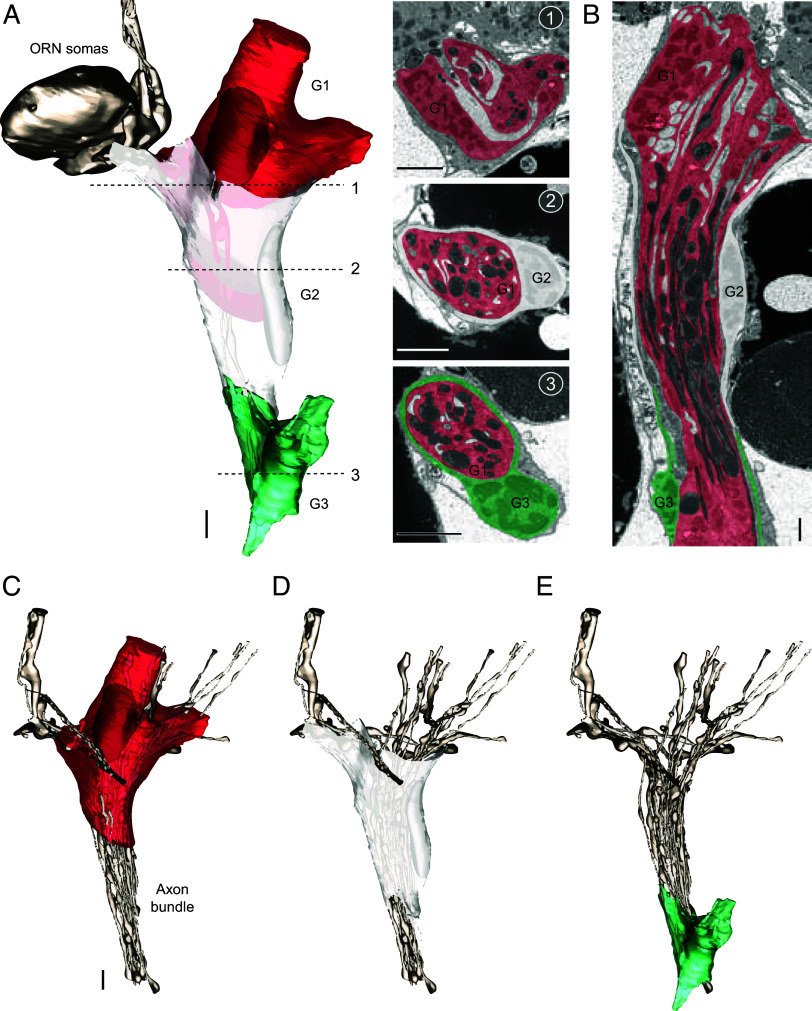
Glial cells associated with cp axons. (*A*) 3D models and SBEM images of a trio of cp axons with their associated glial cells. Cells are pseudocolored to indicate identities: ORNs (bronze), and glial cells (red, white, and green). The ORN models are the same as in Figs. 1 and 3. Three glial cells (G1, G2, and G3) were segmented for 3D reconstruction. For G1, only its outer boundary was segmented; its processes surrounding the axons are shown pseudocolored in the SBEM images. Dashed lines indicate the locations of the corresponding SBEM images. Scale bars: 2 μm for 3D models and 1 μm for SBEM images. (*B*) A longitudinal section rendered by IMOD. (*C*–*E*) 3D models of individual glial cells, showing their relative positions. 3D models of axons within the same nerve fascicle are also shown for reference. Individual glial processes were not segmented for simplicity. See also *SI Appendix*, Fig. S4 and Movie S5.

Extending from the somas, multiple ORN axons converged into a nerve fascicle, along which we identified eight glial nuclei that were unevenly distributed and more concentrated near the proximal region (*SI Appendix*, Fig. S4*A*). Of these, the three most proximal glial cells were selected for 3D reconstruction (Fig. 5*A*). Due to their complex morphology, fine glial processes were not segmented and therefore not shown in the 3D models.

Among the three segmented glial cells, the first cell (G1, closest to the ORN somas) extended processes that wrapped individual axons, appearing to gather them into a nerve fascicle (Fig. 5 *A*–*C*). Notably, its processes extended beyond the initial axonal segment and overlapped with areas covered by other glial cells (Fig. 5 *A* and *B*). Its nucleus was situated at the fascicle periphery, and the cell itself also formed the outer boundary of the nerve close to the ORN somas (Fig. 5 *A* and *B*). This glial cell’s ensheathment of individual ORN axons resembles that of central glial cells in the *Drosophila* antenna (49). However, the central glial cells in flies do not appear to form the outer layer of fascicles. This suggests the intriguing possibility that the mosquito glial cell (G1) represents a previously unrecognized class of glia in the insect olfactory organ.

Further along the nerve bundle, we identified a second glial cell which surrounded the fascicle and overlapped nearly 50% of the region already covered by the first glial cell. Unlike the first cell, this glia did not ensheathe individual axons but instead primarily formed the fascicle’s outer layer (G2 in Fig. 5 *A*, *B*, and *D*). Moving further along, a third glial cell emerged, covering the next segment with minimal overlap with the second cell. Similar to the second cell, its processes were largely confined to the outer layer, and its nucleus was positioned at the fascicle boundary (G3 in Fig. 5 *A*, *B*, and *E*). Based on these anatomical features, the second and third cells likely correspond to peripheral glia, which are known to form the outer layer of ORN nerve fascicles in *Drosophila* (49). A similar pattern was observed in subsequent glial cells, except for the 6th and 8th cells. Unlike the others, these two glia did not constitute the outer layer but instead extended their processes inside the fascicle to ensheathe individual axons. Their nuclei were situated within the fascicle boundary (*SI Appendix*, Fig. S4*B*, Images 5, 6, 8, and 9), exhibiting features characteristic of central glial cells (49).

### Morphometric Analysis of cp ORNs.

**Fig. 6.**
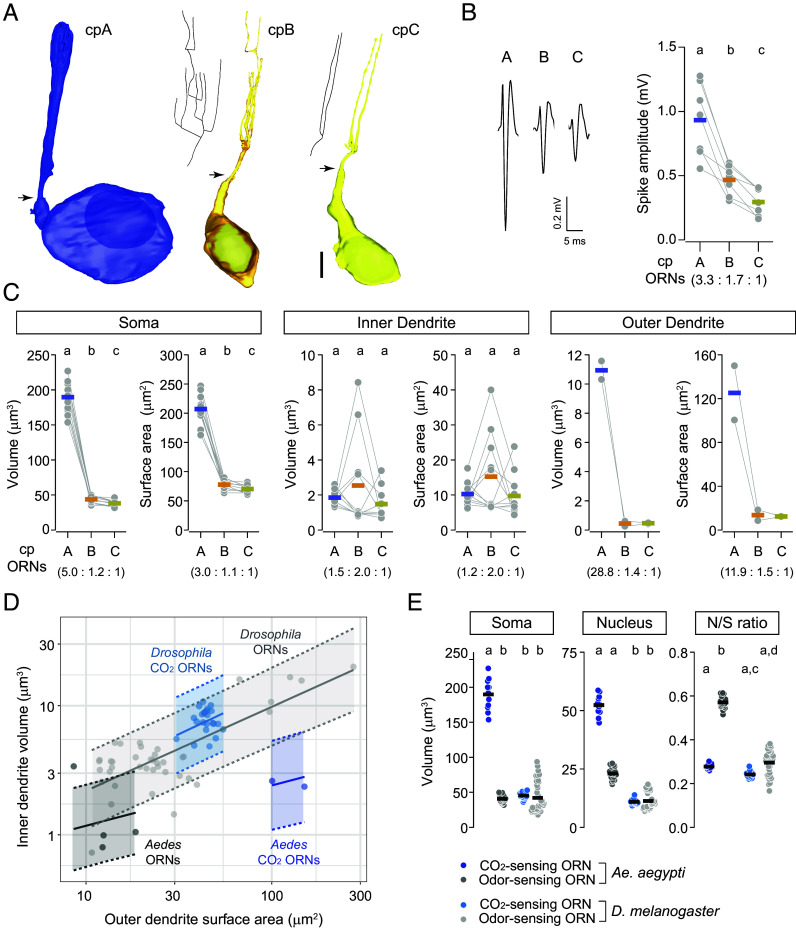
Morphometric analysis of capitate peg ORNs. (*A*) 3D models of cpA, cpB, and cpC neurons. Arrows indicate the locations of ciliary constriction, marking the starting point of the outer dendrite. Nuclei are shown in darker shades. 2D projections of the outer dendritic skeletons are provided for cpB and cpC neurons. (Scale bar: 2 μm.) (*B*) Spike amplitude comparison. (*Left*) Representative spike waveforms for cpA, cpB, and cpC neurons. (*Right*) Quantified spike amplitudes, with each gray point representing an ORN’s average spike amplitude over 10 s. Lines connect measurements from ORNs within the same sensillum, with thick colored lines showing the means. Spike amplitude ratios are relative to the smallest-spike ORN (cpC). *n* = 7 from five mosquitoes. Statistical significance (paired *t* test) is indicated by different letters. (*C*) Morphometric comparison of somatic, inner dendritic, and outer dendritic regions. Lines connect measurements from ORNs within the same sensillum, with thick colored lines showing mean values. Morphometric ratios are relative to the smallest-spike ORN (cpC). *n* = 11 for soma and inner dendrite, and *n* = 2 for outer dendrite. Statistical significance (paired *t* test or Wilcoxon signed-rank test) is indicated by different letters. (*D*) Bayesian regression analysis of log_10_ outer dendritic surface area vs. log_10_ inner dendritic volume. Shaded areas show 90% posterior predictive intervals, and solid lines indicate mean regression slopes. The four groups are the combinations of species (*Aedes* or *Drosophila*) and neuronal type (CO_2_- or odor-sensing ORNs). (*E*) Comparison of soma volume, nucleus volume, and nucleus-to-soma ratio (N/S) across CO_2_- and odor-sensing ORNs in *A. aegypti* and *D. melanogaster*. Statistical significance (Kruskal–Wallis one-way ANOVA on ranks) is indicated by different letters. See also *SI Appendix*, Fig. S5.

For morphometric analysis, we segmented the maximum number of cp ORNs within our SBEM volume, including the nuclei, somata, and inner dendrites of 11 cp ORN trios, as well as the complete outer dendrites of two sets of cp ORNs. The cp outer dendrites that were damaged or truncated during image acquisition were excluded from the survey. Our morphometric analysis thus included two fully reconstructed sets of cp ORNs (Fig. 6*A* and Movie S1) and several partially segmented neurons with only their soma and inner dendrite reconstructed.

Analysis of spontaneous spike activity of grouped cp ORNs revealed their extracellular spike amplitude ratio (3.3:1.7:1, Fig. 6*B*). As described, cp ORNs were distinguished by soma size, with the A neuron being much larger than the B or C neuron, exhibiting a volume ratio of 5.0:1.2:1 and a surface area ratio of 3.0:1.1:1 (Fig. 6 *C*, *Left* and *SI Appendix*, Table S1). However, no significant differences were found in the inner dendritic size (Fig. 6 *C*, *Middle*), unlike *Drosophila* ORNs where inner dendritic size correlates with soma size (28).

Moreover, we observed a striking disparity in the outer dendritic morphometrics of cpA compared to cpB and cpC neurons, with a volume ratio of 28.8:1.4:1 and a surface area ratio of 11.9:1.5:1 (Fig. 6 *C*, *Right*). That is, the CO_2_-sensing cpA neuron had a significantly larger sensory surface area, which may contribute to the CO_2_ acuity of *A. aegypti*. Further, this striking outer dendritic size disparity can also enhance ephaptic inhibition asymmetry (26), making cpA far more dominant than cpB or cpC. This asymmetry is expected to influence mosquito behavioral responses to odor blends containing CO_2_ and odorants that activate cpB and cpC (24).

### Comparison between Mosquito and Fly ORNs.

Given the ethological importance of CO_2_-sensing in insects (2), we wondered how the morphometric features of cp ORNs in *A. aegypti* compare to those of CO_2_- and odor-sensing ORNs in *D. melanogaster*. One key difference is likely the relationship between the outer dendritic (OD) surface area and inner dendritic (ID) volume, which scales with mitochondrial counts in *Drosophila* ORNs (28). Due to the smaller sample size of mosquito cp ORNs compared to their fly counterparts (*SI Appendix*, Table S1), we used Bayesian regression to examine how changes in OD surface area corresponded to changes in ID volume and whether this relationship varied by neuronal types and species (Fig. 6*D*).

The posterior distributions of the regression slopes (log_10_ OD surface area vs. log_10_ ID volume) suggest that within each species, both CO_2_-sensing and odor-sensing ORN types followed similar scaling relationships between OD surface area and ID volume (Fig. 6*D* and *SI Appendix*, Fig. S5 *A* and *B*). However, in flies, a given fold change in OD surface area corresponded to a greater fold change in ID volume than in mosquitoes (Fig. 6*D* and *SI Appendix*, Fig. S5*B*). Of note, when combining the CO_2_- and odor-sensing ORN data for regression analysis (*SI Appendix*, Fig. S5*C*), we found that most of the posterior distribution for the difference between flies and mosquitoes fell above zero, with a minor peak around zero (*SI Appendix*, Fig. S5*D*), suggesting a likely but not definitive difference. To assess the strength of evidence for species-specific differences, we compared models with and without a species-specific slope term, and found that the former predicted the data better than the latter (data not shown). Overall, our analysis supports a significant difference in the relationship between OD surface area and ID volume between fly and mosquito ORNs. These findings raise the possibility that mosquito cpA may have developed adaptations to compensate for the lack of enlarged, mitochondria-rich inner dendrites observed in fly ORNs (28).

Indeed, mosquito cpA neurons had significantly larger cell bodies than cpB, cpC, or any characterized fly ORNs—including CO_2_-sensing ab1C neurons and a variety of odor-sensing ORN types (27, 28) (Fig. 6 *E*, *Left*). Given cpA’s numerous somatic mitochondria (Fig. 1*E*, Image 13), its exceptionally large soma likely evolved to meet the high metabolic demands of an extensive sensory surface.

Furthermore, mosquito cp ORNs had markedly larger nuclei than their fly counterparts (Fig. 6 *E*, *Middle*). Specifically, cpB and cpC neurons had a much higher nucleus-to-soma volume ratio (0.52 to 0.61), compared to 0.26 to 0.30 in cpA and 0.17 to 0.38 in fly ORNs (Fig. 6 *E*, *Right*). This high nucleocytoplasmic ratio in cpB and cpC raises intriguing questions for future research, such as whether these postmitotic neurons have reduced nucleocytoplasmic transport or altered chromatin–cytoskeleton interactions (50, 51).

### A Possible Anatomical Adaptation for Enhancing CO_2_ Accessibility.

In mosquitoes, the cell bodies of cp ORNs were positioned more superficially than those of the CO_2_-sensing ab1C and odor-sensing ab1D in flies, located at 5.98 µm vs. 10.37 µm below the cuticle base (*SI Appendix*, Fig. S6 *A* and *B*, ab1 data from ref. 27). As a result, 82% of the mosquito cpA outer dendritic length was encapsulated within the sensillum cuticle above the base, compared to 62% for fly ab1C (*SI Appendix*, Fig. S6*C*). Of note, in both species, a significant portion of the outer dendrite beneath the cuticle base (i.e., not encapsulated by the sensillum cuticle) is enveloped by the thecogen cell (27, 28) (Fig. 4*C*), occluding the ensheathed region from odor exposure. Therefore, this greater proportion of sensillum-encapsulated outer dendrite may represent an anatomical adaptation that enhances the accessibility of the mosquito cpA sensory surface to CO_2_.

## Discussion

This study provides a detailed 3D characterization of CO_2_-sensing cpA neurons in *A. aegypti* by uncovering their morphological specializations. The markedly enlarged dendritic surface area of cpA neurons, shaped by intricate lamellar folding, likely facilitates CO_2_ detection. Further, compared to odor-sensing cpB and cpC neurons, cpA neurons exhibit a strikingly larger outer dendritic size, a morphometric disparity which is expected to contribute to cpA’s ephaptic dominance within the cp sensillum and may influence how mosquitoes integrate CO_2_ and other host-derived odor cues. Moreover, the greater encapsulation of mosquito cpA dendrites within the sensillum cuticle, compared to CO_2_-sensing ab1C neurons in *Drosophila*, suggests an anatomical adaptation in mosquitoes that enhances CO_2_ accessibility. Our comparative analysis of *Aedes* and *Drosophila* offers valuable evolutionary insights into orthologous sensory neurons in two distinct Dipteran lineages—*Nematocera and Brachycera*—by providing evolutionary morphological comparison at the cellular level, and by revealing how orthologous neurons can diverge to meet distinct ecological demands.

Using SBEM with cryofixation, we also performed nanoscale morphometric measurements of mosquito neurons, generating high-quality data for biorealistic modeling of how dendritic architecture affects chemosensory function in future studies. Of note, a flattened dendritic sheet has lower input resistance than a cylindrical dendrite of the same cross-sectional area, as its larger surface area facilitates greater ionic flow through the membrane. Additionally, cpA’s thick, trunk-like proximal dendrite reduces axial resistance compared to the much narrower dendrites of odor-sensing cpB and cpC neurons (Fig. 1*E*, Image 10). Together, these morphological features are expected to permit cpA-specific signal conduction, potentially enhancing the neuron’s electrical responsiveness to CO_2_ at the sensory dendrites.

Another distinctive feature of cpA is its axon, which is decorated with numerous mitochondria-enriched varicosities. While a “pearls-on-a-string” morphology is not uncommon—being broadly observed in unmyelinated axons of mouse hippocampal neurons and attributed to biophysical forces from membrane mechanics—these structures do not contain a higher density of mitochondria (46). Hippocampal CA3-to-CA1 presynaptic boutons and autonomic neuroeffector junctions also exhibit axonal varicosities, which contain numerous synaptic vesicles and often multiple mitochondria (52, 53). In stark contrast, cpA axonal varicosities lack synaptic vesicles but are still filled with mitochondria (Fig. 3). If these mitochondria are not required for providing energy for synaptic vesicle release, what then might be their functional role in cpA axons?

One possibility is that these mitochondria supply ATP necessary for maintaining ion gradients that sustain action potential generation, while also managing reactive oxygen species produced during neuronal activity. In addition, mitochondria can serve as high-capacity Ca^2+^ buffers in neurons, sequestering Ca^2+^ that rises sharply during high-frequency firing (40, 54–56). A notable consequence of mitochondrial Ca^2+^ uptake is the shortening of the slow afterhyperpolarization phase during such activity (56), which in turn reduces the likelihood or extent of spike frequency adaptation in response to sustained stimuli (57). Supporting this idea, analysis of a single-nucleus transcriptomic atlas of *A. aegypti* revealed high expression of the Ca^2+^-activated potassium channel *slowpoke* (slo, AAEL018306) in cpA neurons (58). Given that the channel mediates slow afterhyperpolarization (59), it raises the possibility that the mitochondria-enriched cpA axonal varicosities help minimize spike frequency adaptation during prolonged exposure to host-emitted CO_2_.

Beyond these potential functional benefits, cpA’s pearls-on-a-string axons could entail functional compromises. Computation modeling suggests that such geometrical irregularities would slow down action potential velocity and delay propagation (60, 61). Moreover, the occupation of axoplasmic space by mitochondria is expected to increase the internal resistance to current flow, further reducing conduction speed and causing significant spiking delays (62). These findings highlight a potential trade-off between metabolic support and signal transmission efficiency, underscoring the need for future studies to investigate the functional advantages and disadvantages of this unique axonal structure. Also warranting exploration is the possibility that these varicosities function as specialized microdomains, with ionic or protein compositions distinct from other axonal regions.

In addition to these structural differences, our study highlights distinctive cellular features of mosquito cp ORNs, including the large cpA soma, the high nucleus-to-soma ratios in cpB and cpC neurons, and the unique morphology of the tormogen cell and the dedicated glial cell within the cp sensillum. These characteristics suggest that mosquito ORNs have evolved specific metabolic and structural adaptations to support their essential role in host-seeking. The specific ensheathment of the cpA soma by the tormogen and dedicated glial cells raises intriguing questions about its potential functions in neuronal insulation and functional support. Moreover, the absence of a positive correlation between outer dendritic surface area and inner dendritic volume in mosquito ORNs, unlike in flies, underscores species-specific differences that may shape sensory processing in mosquitoes.

Our study also lays the groundwork for future comparative analyses of cpA neurons across mosquito genera, such as *Culex quinquefasciatus* and *A. gambiae*, to explore evolutionary adaptations of CO_2_-sensing neurons. These mosquito species exhibit distinct CO_2_ response properties compared to *A. aegypti* (63, 64), raising the possibility that interspecies differences in sensory physiology may in part arise from structural variations in cpA neurons. Determining which features are conserved versus uniquely specialized could help link morphology to function and clarify the evolutionary significance of the adaptations observed in *A. aegypti*.

Together, these findings provide insights into the anatomical specializations of mosquito CO_2_ detection and signal conduction, paving the way for upcoming investigations to examine the functional significance of these unique structures and the molecular or developmental programs that shape ORN morphology.

## Materials and Methods

### Animals.

*A. aegypti* mosquitoes (Liverpool strain) were reared under controlled conditions in incubators set to 28 °C, 40% relative humidity, and a 12-h light/dark cycle. Adults were housed in Bugdorm cages (24.5 × 24.5 × 24.5 cm) with continuous access to 10% (w/v) sucrose solution. To sustain the mosquito colony, females were provided a blood meal from anesthetized mice for approximately 15 min, after which oviposition substrates were introduced 3 d later. Eggs were allowed to melanize for 2 d before being floated in trays for hatching. Larvae were reared in plastic containers (Sterilite, 34.6 × 21 × 12.4 cm, USA) containing approximately three liters of deionized water and were fed a mixture of ground TetraMin fish food and yeast powder.

#### Ethical conduct of research.

Mice used for mosquito blood feeding were handled in accordance with the Guide for the Care and Use of Laboratory Animals as recommended by the NIH and approved by the UCSD Institutional Animal Care and Use Committee (Animal Use Protocol #S17187) and UCSD Biological Use Authorization (BUA #R2401).

For both SBEM imaging and single-sensillum recordings, 3- to 5-d-old female mosquitoes were used. The mosquitoes were anesthetized on ice, had their wings clipped under a stereomicroscope, and then transferred to the EM facility or to the lab for electrophysiological recordings.

### Tissue Preparation and SBEM Volume Acquisition.

For SBEM experiments, the mosquitoes were anesthetized on ice, and their maxillary palps were removed by pinching the first or second palp segment with fine forceps. The SBEM volume of the *A. aegypti* maxillary palp was generated following the CryoChem protocol. Briefly, dissected olfactory tissues were immediately subjected to high-pressure freezing, followed by freeze-substitution, rehydration, en bloc heavy metal staining, dehydration, and resin infiltration, as described (31). Microcomputed X-ray tomography was used to determine the position and proper orientation of the resin-embedded specimens. Samples were mounted on aluminum pins with conductive silver epoxy and sputter coated with gold-palladium for SBEM imaging with a Gemini SEM 300 (Zeiss) equipped with a Gatan 3View 2XP microtome system and the OnPoint backscatter detector.

The antennal SBEM volume was acquired at 2.5 kV using a 30-μm aperture, with the electron gun set to analytic mode and the beam operating in high-current mode. Nitrogen gas was used for focal charge compensation to reduce charging artifacts. Imaging was performed with a dwell time of 1 μs, a pixel size of 5 nm, and a Z-step of 40 nm. The X and Y pixel numbers were 1,733 and 1,549, respectively, and there were a total of 2,942 Z slices. After data collection, the images were converted to MRC format, and rigid alignment of the image slices was performed using cross-correlation in the IMOD image processing package (https://bio3d.colorado.edu/imod/). The SBEM volume is available in the Cell Image Library (https://www.cellimagelibrary.org/) with the accession number CIL:57520.

### Image Segmentation.

In the mosquito palp volume, the cp sensilla were identified as the only palp olfactory sensilla containing three neurons. These sensilla are characterized by their club-shaped cuticle and the presence of three ORNs (30). Manual segmentation was conducted using the IMOD software (https://bio3d.colorado.edu/imod/) (65) with the drawing tools by placing closed contours around the structures of interest in serial sections. The sensillum cuticle, ORN soma, and inner and outer dendritic segments were saved as distinct objects to facilitate morphometric measurement of individual structures. The ciliary constriction was used to define the boundary between the inner and outer dendrites (47).

For the cpA neurons, which have extensively lamellated outer dendrites, each dendritic lamella was segmented as an individual object. All segmented objects were then “meshed” to connect adjacent contours to form continuous 3D structures. Detailed information about “imodmesh” and IMOD’s drawing tools is available in the IMOD user guide (https://bio3d.colorado.edu/imod/doc/man/imodmesh.html; https://bio3d.colorado.edu/imod/doc/3dmodHelp/plughelp/drawingtools.html).

### SBEM Image Postprocessing.

For representative SBEM images, image quality was enhanced using the DenoiseEM plug-in for ImageJ, which offers multiple denoising algorithm options. Briefly, TIFF images were first loaded into ImageJ and converted to a 16-bit file format. Multiple regions of interest within the sensillar lumen were sampled to train the denoising algorithms, and the optimal algorithm was selected based on the best signal-to-blur ratio or overall image quality. For the SBEM images presented in this study, the Gaussian algorithm was most frequently used. To further enhance the visibility of dendritic branches, the contrast and brightness of the denoised images were adjusted in ImageJ. The final images were then converted back to RGB format and exported as TIFF files. Detailed information about DenoiseEM is available in the DenoiseEM plug-in page (https://bioimagingcore.be/DenoisEM/).

### Skeletonization.

To visualize the dendritic branching patterns of cpB and cpC neurons, the 3D models of ORN dendrites in MOD format were first converted to VRML2 files using the command “imod2vrml2” in IMOD. The VRML2 files were then imported into Amira (2020.2 version; ThermoFisher Scientific, USA) and converted into a binary volume, with the 3D model area colored in white and the background in black. The AutoSkeleton module in Amira was used to generate skeletons of the dendrites, which were then manually edited using the “Filament editor” in Amira by overlaying them with ORN 3D models to correct errors such as extra loops or branches.

These skeletons, in SWC format, were imported into neuTube (https://www.neutracing.com/), where dendritic branches were manually spread onto a 2D plane. Briefly, a primary branch and all its downstream branches were first selected to allow all the branches to be edited and moved as a group. This process was repeated for secondary, tertiary, and higher-order branches until overlap between branches was minimized.

### Morphometric Analysis.

For morphometric analysis, the sensillum cuticle, ORN soma, inner dendrite, and the proximal and distal outer dendritic segments were analyzed as separate objects.

#### Surface area and volume.

The morphometric values were extracted from individual objects using the “imodinfo” function in IMOD. Detailed information about imodinfo is available in the IMOD User’s Guide (https://bio3d.colorado.edu/imod/doc/man/imodinfo.html). The total volume and surface area of the dendritic lamellae in cpA neurons, as well as the dendritic branches in cpB and cpC neurons, were calculated by summing the measurements from each individual lamella or branch.

#### Length.

To calculate the lengths of individual objects, the structures were first skeletonized using Amira. The resulting SWC files were imported into R, where pixel coordinates were scaled to micrometers using scaling factors derived from the “imodinfo” command. The length of each component was then calculated using the Pythagorean theorem.

#### Axon varicosity.

Axon morphologies were initially converted from .mod to .vrml2 format. The resulting vrml2 files were imported into Amira to generate two key data structures: 1) A binary volume, a 3D image stack representing the axon surface, where voxels inside the surface were assigned a value of 1, and those outside a value of 0; and 2) An axon skeleton in SWC format, computed using Amira’s AutoSkeleton module and manually smoothed to reduce sharp corners.

The binary volume was transformed into a point cloud by extracting the XYZ coordinates of all voxels with a value of 1. Each point in this cloud was then orthogonally projected onto the nearest location along the axon skeleton. These projection positions were normalized to a 0 to 1 scale, representing their relative location along the axon’s length.

A histogram (bin size = 0.001) of the normalized positions was created to quantify the local density of surface points along the axon. The normalized positions were then scaled by the physical axon length (µm) to generate x-axis values in micrometers. The y-axis, representing local cross-sectional area (µm^2^), was computed by scaling the histogram frequencies: first normalizing them so the total area under the curve equaled 1, then multiplying by the total axon volume (µm^3^) to ensure correct physical units. This resulted in a curve whose summed area corresponds to the total axonal volume.

To reduce noise, the cross-sectional area curve was smoothed using a 1D Gaussian filter (gaussian_filter1d) from the scipy.ndimage package. Peaks in the smoothed curve (y_smooth) were identified as “varicosities” using the find_peaks function from scipy.signal, with a minimum prominence threshold of 30% of the maximum y-value (excluding outliers). Varicosity lengths were defined as the full width at half maximum of each peak, calculated using peak_widths. The regions between varicosities, designated as “connectors,” had their cross-sectional areas defined as the mean y_smooth values across their respective intervals.

#### Axonal mitochondria.

Mitochondria within each axon were processed using the same pipeline as the axons described above. Their segmented and binarized volumes were converted into point clouds, projected onto the axon skeletons, normalized, histogrammed, and smoothed using a Gaussian filter, following the same steps outlined for axons. To compute mitochondrial occupancy, the smoothed mitochondrial cross-sectional area curve was divided elementwise by the corresponding axonal cross-sectional area curve. The result was a dimensionless occupancy profile that describes the relative spatial distribution of mitochondria along each axon.

#### Soma position.

To calculate the depth of the ORN soma center of mass (COM) below the cuticle base, the process involved two main steps. First, the cuticle proportion of the soma COM (*C*) was calculated. The SWC skeletons of sensillum cuticle and ORN soma were first imported into Python. The soma COM was projected onto the nearest point on the cuticle skeleton. The cuticle proportion was defined as the relative position of the soma COM along the cuticle, scaled from 0 at the cuticle base to 1 at the cuticle tip. In cases where the soma COM was located below the cuticle base, the bottom segment of the cuticle skeleton was extrapolated, and the soma COM was projected onto this extrapolated segment. For these cases, the cuticle proportion (*C*) was scaled from 0 at the cuticle base to negative infinity, with a value of −1 indicating the soma COM was one cuticle length below the extrapolated segment.

Next, the depth of the soma COM below the cuticle (*D*) was calculated using the formula:

D=-C×L,

where *L* represents the total cuticle length in µm and *C* is the previously calculated cuticle proportion of the soma COM. The depth *D* was then classified: If *D* was greater than 0, the soma COM was located below the cuticle base; if *D* equaled 0, it was at the cuticle base; and if *D* was less than 0, the soma COM was above the cuticle base, which typically indicated that the soma was laterally placed (see *SI Appendix*, Fig. S2*B* for illustrations).

*Proportion of the outer dendrite encapsulated in the sensillum cuticle:* The proportion of the outer dendrite covered by the sensillum cuticle was measured to assess how much of the dendrite has direct access to CO_2_ or odorants. To calculate cuticle coverage, the cuticle base was projected onto the closest point on the outer dendrite skeleton. Coverage was defined as the relative position of this point along the outer dendrite, ranging from 0, where the cuticle base was projected to the tip of the outer dendrite, to 1, where it was projected to the position of ciliary constriction.

#### Single-sensillum recording and spike analysis.

Mosquitoes were briefly cold-anesthetized on ice before their legs and proboscis were removed. Their bodies were then placed on double-sided tape affixed to a coverslip. The maxillary palps were stabilized using a short piece of human hair. To record the extracellular electrical activity of capitate peg ORNs, a sharp aluminosilicate glass electrode filled with adult hemolymph-like (AHL) solution (66) was inserted into a sensillum, while a reference electrode, also filled with AHL solution, was placed in the eye. No more than three sensilla were recorded from a mosquito. Alternating current signals (band-passed: 100 to 20,000 Hz) were recorded using an NPI EXT-02F amplifier (ALA Scientific Instruments) and digitized at 5 kHz with a Digidata 1550 (Molecular Devices). ORN spikes were sorted and analyzed offline using Clampfit 10 (Molecular Devices) and Igor Pro (WaveMetrics).

### Bayesian Regression Modeling.

Two alternative hierarchical Bayesian regression models were fitted to the data. In the full model, the log_10_-transformed inner dendritic volume (*y*) was modeled as a linear function of the log_10_-transformed outer dendritic surface area (*x*). To account for the multilevel structure of the data and to stabilize parameter estimates for the mosquito data, which had a small sample size, the model was organized hierarchically.

Data points were assumed to follow a normal distribution around a predicted value (*μi*), which was derived from a linear model with group-specific intercepts (*α_g_*) and slopes (*β_g_*). The groups were defined by the four combinations of species (*Aedes* or *Drosophila*) and neuronal type (CO_2_-sensing or odor-sensing ORNs). The group-specific intercepts were represented as the sum of an overall intercept (*α_overall_*) and a deviation, with a noncentered parameterization used to improve Markov Chain Monte Carlo (MCMC) sampling efficiency. The group-specific slopes were modeled as the sum of an overall slope (*β_overall_*), species-specific deviations (*δ_s_*) from this overall slope, and group-specific deviations within species (*γ_g_*).

The prior distribution for the overall slope was centered at 3/2, based on the reasoning that the volume of an object scales with the cube of its size, while surface area scales with the square. Slope terms were constrained to be nonnegative, and weakly informative priors were used. Sensitivity analyses indicated that model estimates were robust to variations in the hyperparameters of the priors.

The full model is

$$y_{i}∼N\left(\mu_{i},\sigma_{\mathrm{observation}}\right),$$

$$\sigma_{\mathrm{observation}}∼{\mathrm{Cauchy}}^{+}\left(0,15\right),$$

$$\mu_{i}=\alpha_{g\left(i\right)}+\beta_{\left[g\left(i\right)\right]}\;x_{i}\mathrm{where}\;g\left(i\right)\;\mathrm{is}\;\mathrm{the}\;\mathrm{group}\;\mathrm{for}\;\mathrm{observation}\;i,$$

$$\alpha_{g}=\alpha_{\mathrm{overall}}+\sigma_{\mathrm{intercept}}z_{g},$$

$$\alpha_{\mathrm{overall}}∼N\left(0,20\right),\sigma_{\mathrm{intercept}}∼{\mathrm{Cauchy}}^{+}\left(0,10\right),z_{g}∼N\left(0,1\right),$$

$$\beta_{g}=\beta_{\mathrm{overall}}+\delta_{s\left(g\right)}+\gamma_{g}\;\mathrm{where}\;s\left(g\right)\;\mathrm{is}\;\mathrm{the}\;\mathrm{species}\;\mathrm{for}\;\mathrm{group}\;g,$$

$$\beta_{\mathrm{overall}}∼N\left(1.5,10\right),\delta_{s}∼N\left(0,\sigma_{\delta_{s}}\right),\sigma_{\delta_{s}}∼{\mathrm{Cauchy}}^{+}\left(0,50\right).$$

To assess the strength of evidence for species-specific differences in regression slopes, the full model was compared to an alternative model that excluded the species-specific slope deviation (*δ_s_*). The expected log-predictive density of both models was evaluated using leave-one-out cross-validation (67) in the R package version 2.8.0 (https://mc-stan.org/loo/).

Posterior distributions were estimated using MCMC sampling in Stan (Stan Development Team, 2024. Stan Reference Manual, v2.36.0. https://mc-stan.org). The sampling process utilized four chains, with a burn-in period of 1,000 steps and a sampling period of 2,000 steps. Model implementation was conducted via the “cmdstanr” package in R (R package version 0.8.1, https://www.R-project.org/; https://discourse.mc-stan.org; https://mc-stan.org/cmdstanr/), and data analysis and visualization were performed using the “tidyverse” suite (68).

### Statistical Analysis.

All values were presented as mean ± SEM unless noted otherwise. Paired *t* tests were used for spike amplitude or morphometric comparisons between neighboring ORNs within the same sensillum if the Shapiro–Wilk normality test was passed. If the normality test was not passed, the Wilcoxon signed rank test was used instead. For comparisons across neuronal types, the Kruskal–Wallis one-way ANOVA on ranks was applied. A *P* value of <0.05 was considered statistically significant.

## Supplementary Material

Appendix 01 (PDF)

Dataset S01 (XLSX)

Movie S1. 3D models of mosquito capitate peg ORNs.

Movie S2. 3D models of cp ORN axons and their mitochondria.

Movie S3. 3D models of auxiliary and glial cells associated with cp ORNs.

Movie S4. Comparison between *Drosophila* and *Aedes tormogen* cells. The 3D model of *Drosophila* tormogen cell is associated with the ab1 sensillum, which also houses the CO_2-_ sensing ab1C ORN. Adapted from (1).

Movie S5. 3D models of glial cells associated with cp axons.

## Data Availability

SBEM images data have been deposited in Cell Image Library (CIL:57520) (34).
