# Supplementary material for: Morphological specializations of mosquito CO2-sensing olfactory receptor neurons
Source: Proc Natl Acad Sci U S A. 2025 Oct 23;122(43):e2514666122. doi: 10.1073/pnas.2514666122 (PMC12582328; doi:10.1073/pnas.2514666122)
Supplement: Supplementary file 1 — Appendix 01 (PDF) [file pnas.2514666122.sapp.pdf]

Supporting Information for
Morphological specializations of mosquito CO_2-sensing olfactory receptor neurons

Shadi Charara[1,5#], Jonathan Choy[1,6#], Kalyani Cauwenberghs[1], Pawel Vijayakumar[1], Renny Ng[1], Keun-Young Kim[2], Shih-Che Weng[3,7], Omar S Akbari[3], Mark H Ellisman[2], Scott A Rifkin[4] and Chih-Ying Su[1*]

Correspondence: Chih-Ying Su
Email: c8su@ucsd.edu

This PDF file includes:

Supporting text
Figures S1 to S6
Tables S1
Legends for Movies S1 to S5
SI References

Other supporting materials for this manuscript include the following:

Movies S1 to S5

Supporting Information Text

Figures

Fig. S1. cpA axons exhibit a pearls-on-a-string morphology

(A) SBEM image showing axons from cpA (blue) and other neurons (bronze) in the same nerve fascicle. Scale bar: 1 μm.

(**B–C**) 3D models of individual cpA axons (**B**) and cpB/C axons (**C**). Axon identities were confirmed by tracing each axon back to its corresponding ORN soma in the SBEM image stack. cpA axons were identified based on their connection to the largest ORN soma. cpB and cpC axons were not distinguished and were grouped together for the subsequent morphometric analysis. Scale bar: 2 μm.

(**D–E**) Dimensions of varicosities (**D**) and connectors (**E**) in cpA and cpB/C axons within the same nerve fascicle. The analysis includes 38 varicosities and 44 connectors from 6 cpA axons, and 83 varicosities and 95 connectors from 12 cpB/C axons. The first varicosity shown in Fig. 3E was excluded from the analysis, as its size exceeded two standard deviations above the mean. Statistical results: $P \leq 0.001$ for both varicosity area and length (cpA vs. cpB/C); $P \leq 0.001$ for connector cross-sectional area, and $P = 0.329$ for connector length.

(**F**) Varicosity density, calculated as the number of varicosity per μm of axon.

Statistical significance was assessed with the Mann-Whitney rank sum test.

Fig. S2. Glia separate ORN somas in fly antenna but not in mosquito maxillary palp

SBEM images of the *Drosophila* antenna (**A**) and *Aedes* maxillary palp (**B**). Representative images show the ORN somatic regions from two distinct olfactory sensilla. In *Drosophila*, glial processes (indicated by white arrowheads) closely envelop neighboring ORN somas and insulate them from each other. These images are taken from our previously published SBEM volume (28). In contrast, in mosquito cp sensilla, glial processes also surround the cpB and cpC somas, but the association is less tight. Intervening glial processes between cpB and cpC somas are rarely observed. In some cp sensilla, the cpB and cpC somas are partially separated by the thecogen cell (shown in pink, Image 2). Scale bar: 1 μm.

Fig. S3. cpA soma is ensheathed by tormogen and a dedicated glial cell

(**A**) 3D models of the cpA neuron and its associated tormogen and glial cells. Cells are pseudocolored to indicate identities: cpA (bronze), tormogen cell (green), and glial cell (crimson). In this view, the glial process lies on top of the tormogen process.

(**B**) Same models as in (**A**), rotated 180º. From this angle, the tormogen process appears on top, covering the glial process.

(**C**) Same models as in (**A**), rotated 90º. This view reveals a small region of the cpA soma not covered by either the tormogen or glial cell (indicated by arrow). Instead, this region is covered by the thecogen cell (pink).
Scale bars: 2 μm for 3D models.

Fig. S4. Uneven distribution of glia along the nerve fascicle
(**A**) 3D models of a trio of cp ORN with their associated glial nuclei. Cells are pseudocolored to indicate identities: ORNs (bronze) and glial cells (red, white, green, and purple). Eight glial nuclei are segmented, indicated as G1 to G8. The ORN models are the same as shown in Fig. 1 and Fig. 3. The first three glial nuclei correspond to the glial cells shown in Fig. 5. Dashed lines indicate the locations of the corresponding SBEM images shown in (**B**). The nerve fascicle is not shown (**A**); instead, it is represented by the ORN axons at the proximal end. Central glial cells (G6 and G8) are shown in purple. The glial processes of G8 could not be reliably identified due to poorer image preservation at this depth. Scale bar: 1 μm for SBEM images.

Fig. S5. Posterior distributions of regression slopes relating $\log_{10}$ outer dendritic surface area to $\log_{10}$ inner dendritic volume
(**A**) Posterior distributions of regression slopes for CO_2-sensing and odor-sensing ORNs in *Ae. aegypti* and *D. melanogaster*. Dashed lines bracket the 90% credible interval, representing the highest posterior density.
(**B**) Posterior distributions of slope differences between ORN types within a species (left panels) and between species for the same ORN type (right panels). Left: A peak around zero (solid red line) suggests no significant difference in regression slopes between ORN types within a species. Right: Peaks above zero indicate likely systematic differences in regression slopes between species.
(**C**) Same as (**A**), but with CO_2-sensing and odor-sensing ORN data combined for regression analysis.
(**D**) Posterior distribution of slope differences between species based on the regression analysis in (**C**). A major peak above zero suggests that *Drosophila* ORNs have a systematically larger slope than *Aedes* ORNs. A minor peak at zero indicates some uncertainty in the difference.
See DatasetS1 for raw data.

Fig. S6. Comparison between mosquito cp and fly ab1 sensilla
(A) 3D models of mosquito cp ORNs (left) and fly ab1 ORNs (right), along with their respective sensillum cuticles (gray). Both sensillum types house CO_2-sensing neurons: cpA (blue), and ab1C (yellow). The enlarged inner dendrites of ab1A (blue) and ab1B (orange) are highlighted by

white arrows. Black arrows indicate ciliary constrictions, marking the beginning of the outer dendrite. White arrowhead marks the base of the sensillum cuticle. Scale bar: 2 μm.

(B) Analysis of ORN soma position beneath the cuticle, as illustrated on the upper right panel. In some mosquito cp ORNs, the soma center-of-mass projection can extend above the level of the cuticle base (lower right panel). Gray dots represent data points from individual neurons, with horizonal bars indicating the mean (*n* = 33 for mosquito cp ORNs; *n* = 39 for fly ab1C and ab1D neurons).

(C) Analysis of the proportion of outer dendrite encapsulated within the sensillum cuticle, as illustrated on the right panel. Gray dots represent data points from individual neurons, with horizonal bars indicating the mean (*n* = 2 for mosquito cpA neurons; *n* = 24 for fly ab1C neurons). Statistical significance was determined using the Mann-Whitney rank sum test.

Fly ab1 ORN models and morphometric data were adapted from (27); see DatasetS1 for raw data.

Movie S1. 3D models of mosquito capitate peg ORNs.

Movie S2. 3D models of cp ORN axons and their mitochondria.

Movie S3. 3D models of auxiliary and glial cells associated with cp ORNs.

Movie S4. Comparison between *Drosophila* and *Aedes* tormogen cells. The 3D model of *Drosophila* tormogen cell is associated with the ab1 sensillum, which also houses the CO_2-sensing ab1C ORN. Adapted from (1).

Movie S5. 3D models of glial cells associated with cp axons.

Dataset S1. Morphometrics source data table.

SI References

1. Choy J, et al. (2025) Population-level morphological analysis of paired CO2- and odor-sensing olfactory neurons in D. melanogaster via volume electron microscopy. *eLife*:RP106389. Available at: https://doi.org/10.7554/eLife.106389.

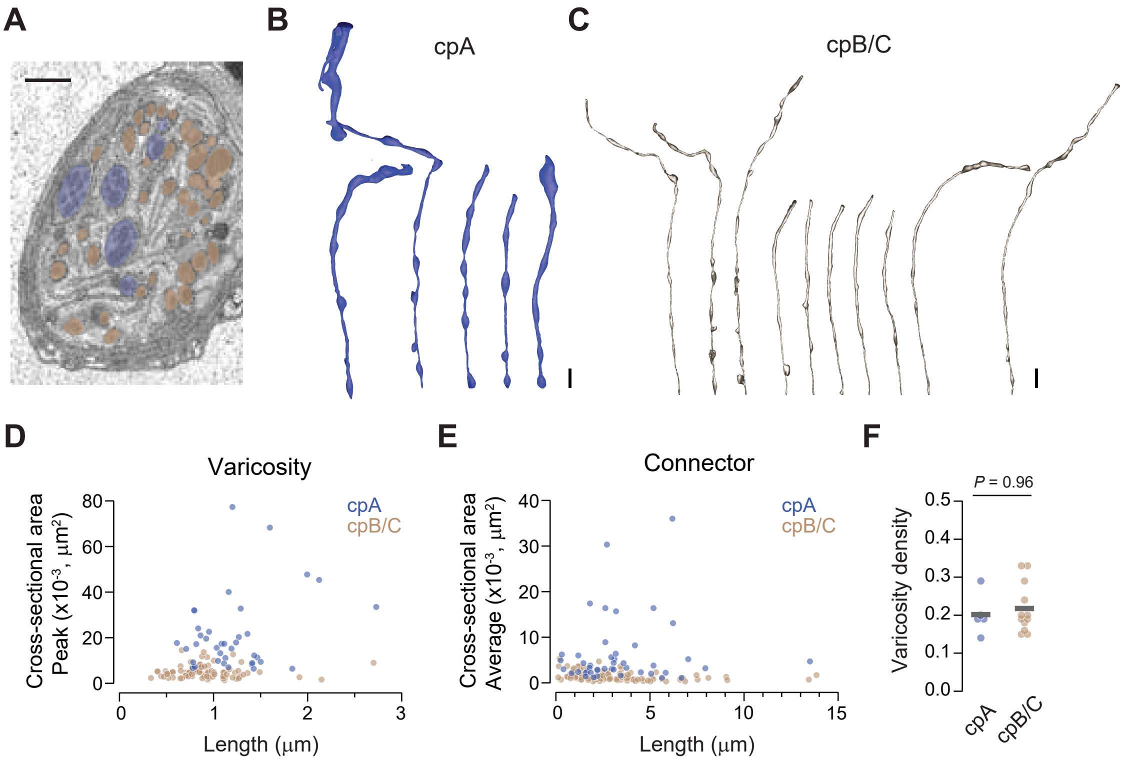

Fig. S1. cpA axons exhibit a pearls-on-a-string morphology

(A) SBEM image showing axons from cpA (blue) and other neurons (bronze) in the same nerve fascicle. Scale bar: 1 μm.

(**B–C**) 3D models of individual cpA axons (**B**) and cpB/C axons (**C**). Axon identities were confirmed by tracing each axon back to its corresponding ORN soma in the SBEM image stack. cpA axons were identified based on their connection to the largest ORN soma. cpB and cpC axons were not distinguished and were grouped together for the subsequent morphometric analysis. Scale bar: 2 μm.

(**D–E**) Dimensions of varicosities (**D**) and connectors (**E**) in cpA and cpB/C axons within the same nerve fascicle. The analysis includes 38 varicosities and 44 connectors from 6 cpA axons, and 83 varicosities and 95 connectors from 12 cpB/C axons. The first varicosity shown in Fig. 3E was excluded from the analysis, as its size exceeded two standard deviations above the mean. Statistical results: $P \leq 0.001$ for both varicosity area and length (cpA vs. cpB/C); $P \leq 0.001$ for connector cross-sectional area, and $P = 0.329$ for connector length.

(**F**) Varicosity density, calculated as the number of varicosity per μm of axon.

Statistical significance was assessed with the Mann-Whitney rank sum test.

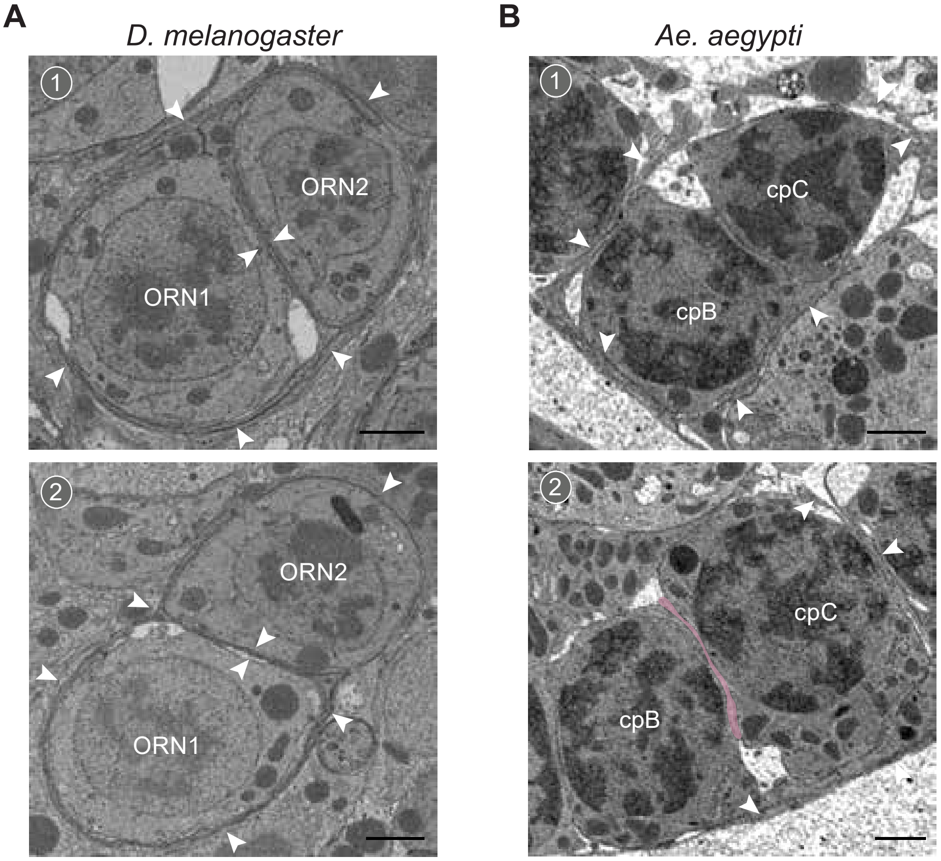

Fig. S2. Glia separate ORN somas in fly antenna but not in mosquito maxillary palp

SBEM images of the *Drosophila* antenna (**A**) and *Aedes* maxillary palp (**B**). Representative images show the ORN somatic regions from two distinct olfactory sensilla. In *Drosophila*, glial processes (indicated by white arrowheads) closely envelop neighboring ORN somas and insulate them from each other. These images are taken from our previously published SBEM volume (28). In contrast, in mosquito cp sensilla, glial processes also surround the cpB and cpC somas, but the association is less tight. Intervening glial processes between cpB and cpC somas are rarely observed. In some cp sensilla, the cpB and cpC somas are partially separated by the thecogen cell (shown in pink, Image 2). Scale bar: 1 μm.

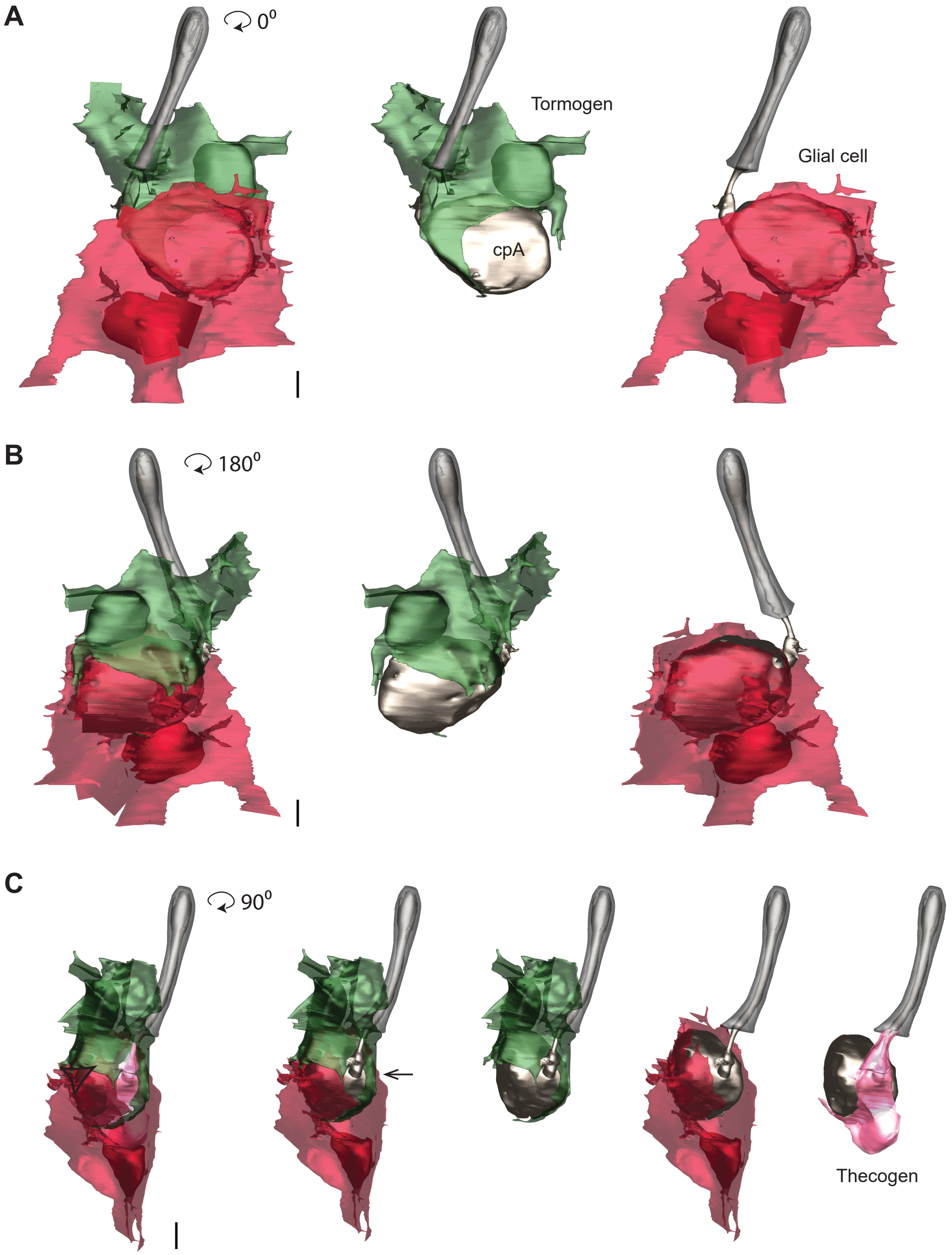

Fig. S3. cpA soma is ensheathed by tormogen and a dedicated glial cell

(**A**) 3D models of the cpA neuron and its associated tormogen and glial cells. Cells are pseudocolored to indicate identities: cpA (bronze), tormogen cell (green), and glial cell (crimson). In this view, the glial process lies on top of the tormogen process.

(**B**) Same models as in (**A**), rotated 180º. From this angle, the tormogen process appears on top, covering the glial process.

(**C**) Same models as in (**A**), rotated 90º. This view reveals a small region of the cpA soma not covered by either the tormogen or glial cell (indicated by arrow). Instead, this region is covered by the thecogen cell (pink).

Scale bars: 2 μm for 3D models.

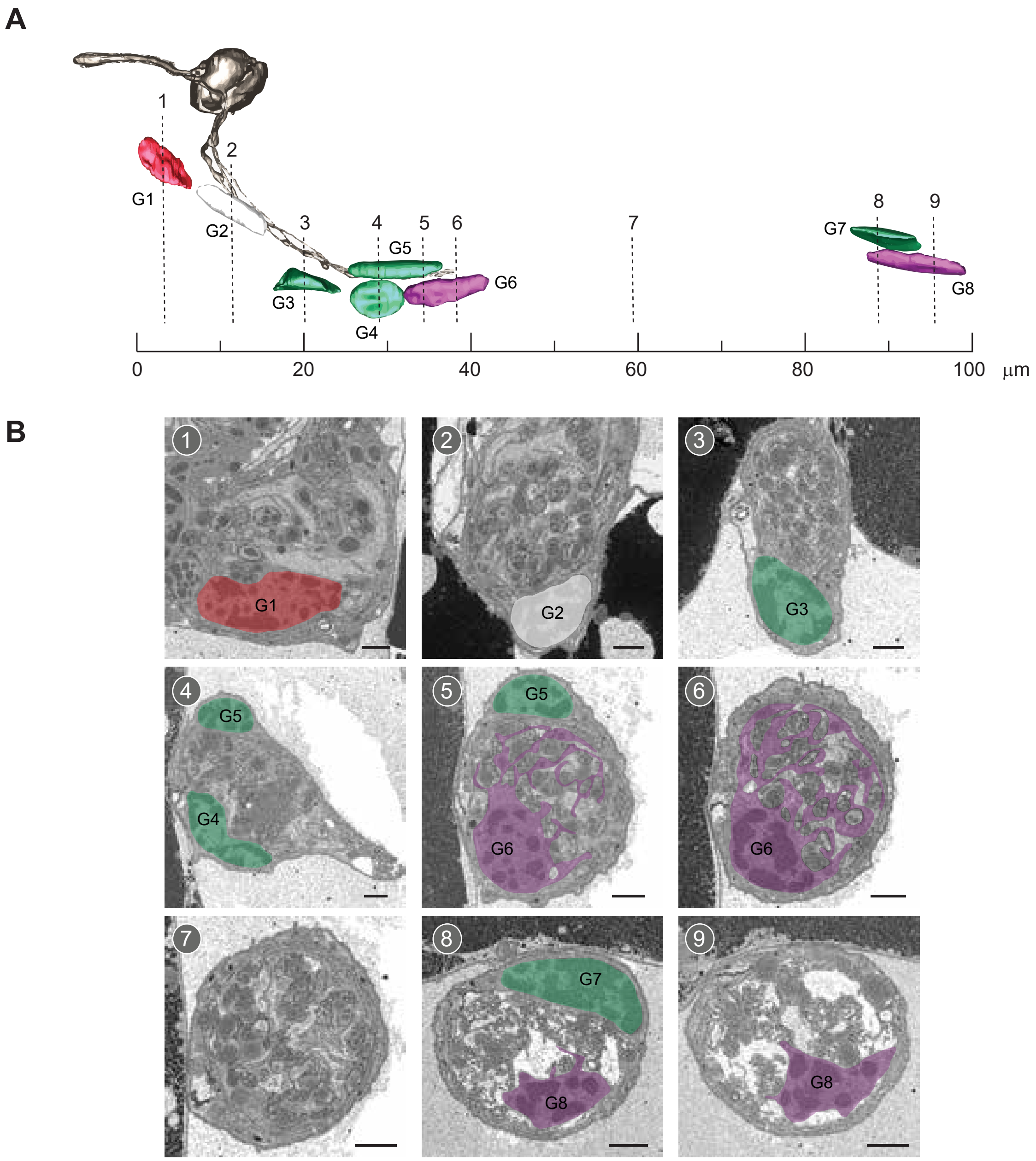

Fig. S4. Uneven distribution of glia along the nerve fascicle

(**A**) 3D models of a trio of cp ORN with their associated glial nuclei. Cells are pseudocolored to indicate identities: ORNs (bronze) and glial cells (red, white, green, and purple). Eight glial nuclei are segmented, indicated as G1 to G8. The ORN models are the same as shown in Fig. 1 and Fig. 3. The first three glial nuclei correspond to the glial cells shown in Fig. 5. Dashed lines indicate the locations of the corresponding SBEM images shown in (**B**). The nerve fascicle is not shown (**A**); instead, it is represented by the ORN axons at the proximal end. Central glial cells (G6 and G8) are shown in purple. The glial processes of G8 could not be reliably identified due to poorer image preservation at this depth. Scale bar: 1 μm for SBEM images.

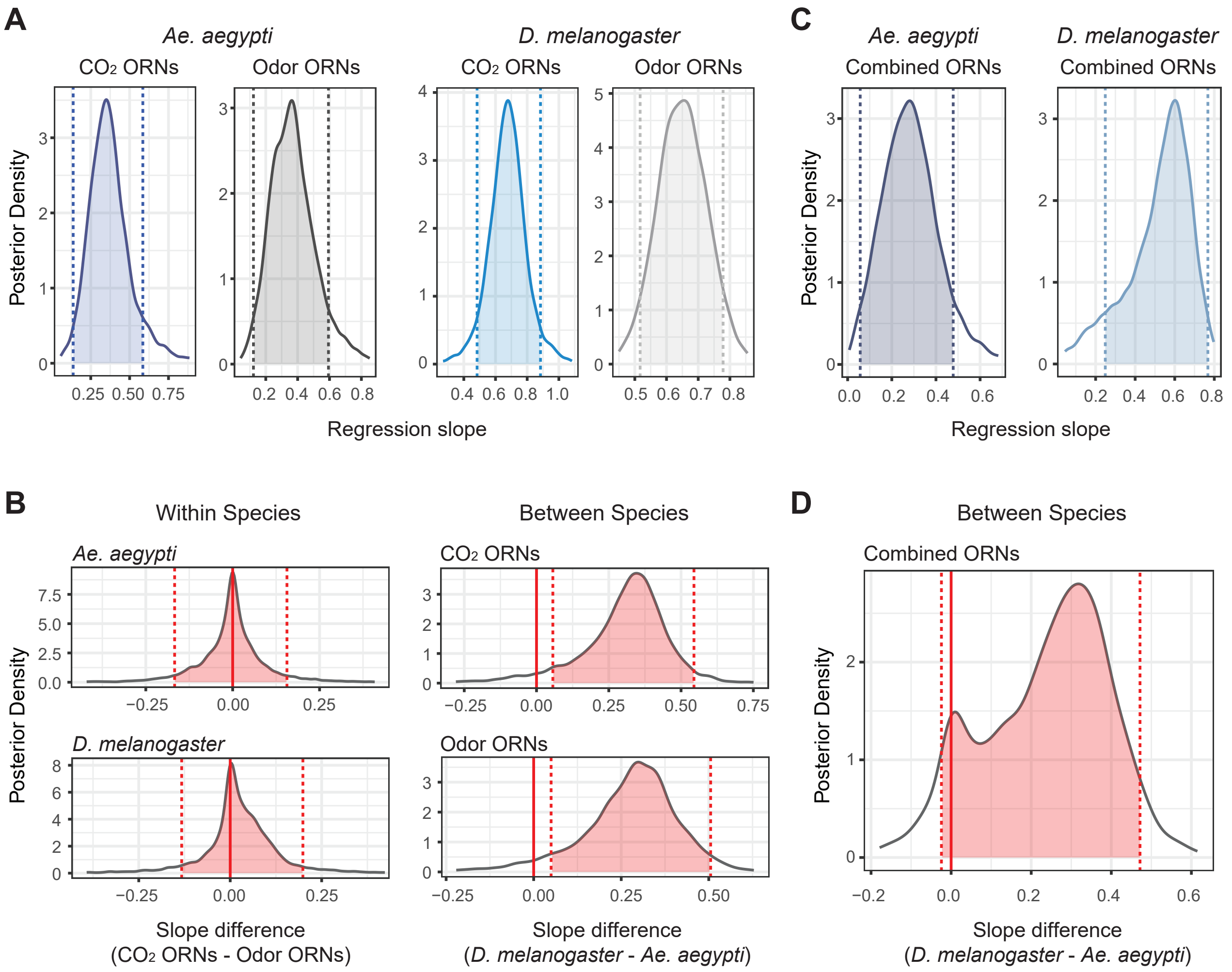

Fig. S5. Posterior distributions of regression slopes relating $\log_{10}$ outer dendritic surface area to $\log_{10}$ inner dendritic volume

(**A**) Posterior distributions of regression slopes for CO_2-sensing and odor-sensing ORNs in *Ae. aegypti* and *D. melanogaster*. Dashed lines bracket the 90% credible interval, representing the highest posterior density.

(**B**) Posterior distributions of slope differences between ORN types within a species (left panels) and between species for the same ORN type (right panels). Left: A peak around zero (solid red line) suggests no significant difference in regression slopes between ORN types within a species. Right: Peaks above zero indicate likely systematic differences in regression slopes between species.

(**C**) Same as (**A**), but with CO_2-sensing and odor-sensing ORN data combined for regression analysis.

(**D**) Posterior distribution of slope differences between species based on the regression analysis in (**C**). A major peak above zero suggests that *Drosophila* ORNs have a systematically larger slope than *Aedes* ORNs. A minor peak at zero indicates some uncertainty in the difference.

See Dataset S1 for raw data.

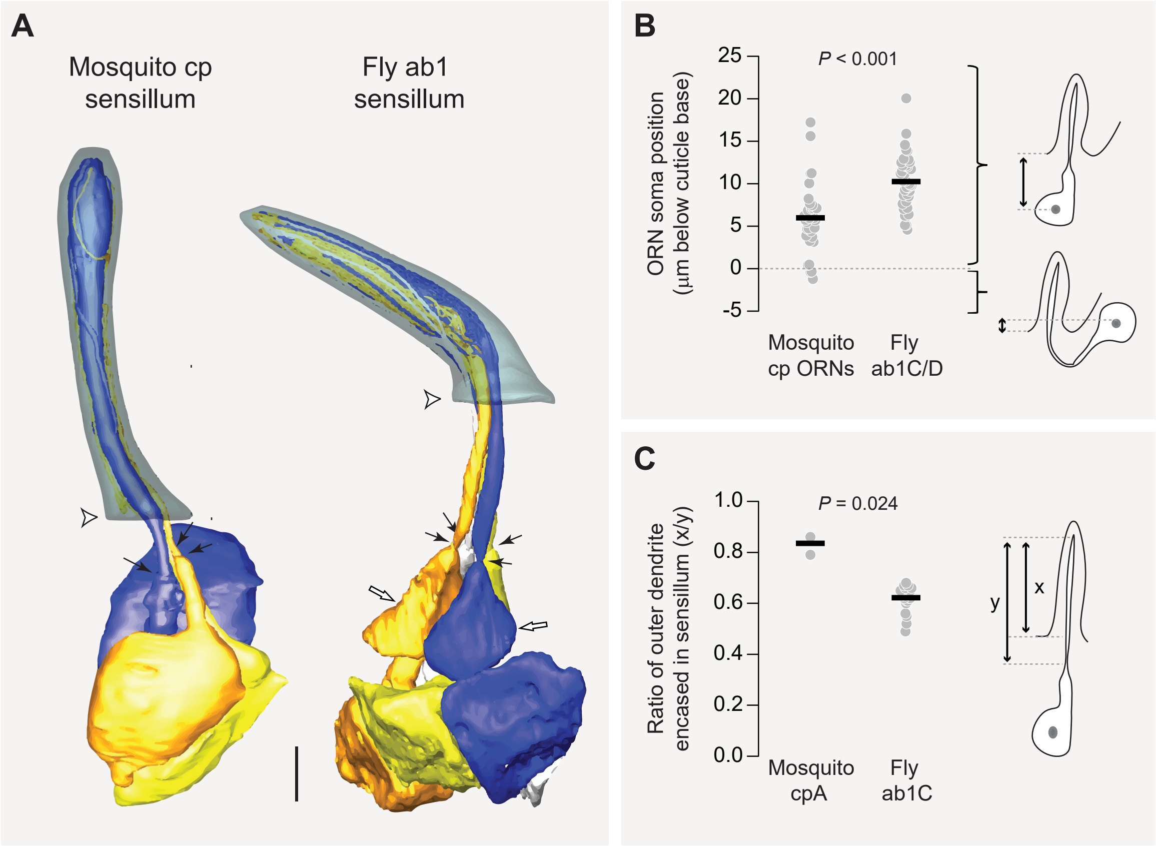

Fig. S6. Comparison between mosquito cp and fly ab1 sensilla

(A) 3D models of mosquito cp ORNs (left) and fly ab1 ORNs (right), along with their respective sensillum cuticles (gray). Both sensillum types house CO_2-sensing neurons: cpA (blue), and ab1C (yellow). The enlarged inner dendrites of ab1A (blue) and ab1B (orange) are highlighted by white arrows. Black arrows indicate ciliary constrictions, marking the beginning of the outer dendrite. White arrowhead marks the base of the sensillum cuticle. Scale bar: 2 µm.

(B) Analysis of ORN soma position beneath the cuticle, as illustrated on the upper right panel. In some mosquito cp ORNs, the soma center-of-mass projection can extend above the level of the cuticle base (lower right panel). Gray dots represent data points from individual neurons, with horizonal bars indicating the mean (n = 33 for mosquito cp ORNs; n = 39 for fly ab1C and ab1D neurons).

(C) Analysis of the proportion of outer dendrite encapsulated within the sensillum cuticle, as illustrated on the right panel. Gray dots represent data points from individual neurons, with horizontal bars indicating the mean (n = 2 for mosquito cpA neurons; n = 24 for fly ab1C neurons). Statistical significance was determined using the Mann-Whitney rank sum test.

Fly ab1 ORN models and morphometric data were adapted from (27); see Dataset S1 for raw data.
